# Chromothripsis in lipoblastoma: second reported case with complex *PLAG1* rearrangement

**DOI:** 10.1186/s13039-023-00665-x

**Published:** 2023-11-27

**Authors:** Joel Lanceta, Joseph Tripodi, Lynne Karp, Meira Shaham, Nayyara Mahmood, Vesna Najfeld, Morris Edelman, Ninette Cohen

**Affiliations:** 1 Department of Pathology and Laboratory Medicine, Northwell Health/Donald and Barbara Zucker School of Medicine at Hofstra, Manhasset, New York USA; 2 Tumor CytoGenomics, Department of Pathology, Icahn School of Medicine at Mount Sinai, New York, NY USA; 3 Division of Cytogenetics and Molecular Pathology, North Shore University Hospital, Manhasset, NY USA; 4 Division of Pediatric Pathology, Cohen Children’s Medical Center, New Hyde Park, NY USA

**Keywords:** Chromothripsis, Lipoblastoma (LPB), FISH, Microarray, Chromosomes, *PLAG1*

## Abstract

Lipoblastomas (LPBs) are rare benign neoplasms derived from embryonal adipose that occur predominantly in childhood. LPBs typically present with numeric or structural rearrangements of chromosome 8, the majority of which involve the pleomorphic adenoma gene 1 (*PLAG1*) proto-oncogene on chromosome 8q12. Here, we report on a LPB case on which showed evidence of chromothripsis. This is the second reported case of chromothripsis in LPB.

## Background

LPBs are rare benign neoplasms derived from embryonal adipose tissue first described more than seven decades ago [1–3]. They are usually diagnosed in children during the first three years of life but can also occur rarely in older children and adolescents. LPBs can occur localized or in a diffuse presentation (lipoblastomastosis) that is extensive and infiltrative [4]. Most LPBs occur in subcutaneous tissue of the trunk and extremities, although they can occur elsewhere in the body. The histomorphology is highly diverse, usually showing a spectrum of lipoblasts, immature and mature adipocytes, primitive mesenchymal cells with a variable myxoid stroma and delicate vasculature. Treatment is complete surgical removal; however, LPBs have been seen to recur in 13–46% of cases due to incomplete excision [5].

LPBs typically present with numeric or structural rearrangements of chromosome 8. Most chromosomal aberrations seen in LPBs involve the *PLAG1* gene located at chromosome 8q12. *PLAG1* is a proto-oncogene activated in other benign and malignant neoplasms, pleomorphic adenoma of the salivary gland, hepatoblastoma and uterine leiomyosarcoma [6–8]. The expression of *PLAG1* is typically upregulated in LPB, most commonly via gene rearrangements; the resulting promoter swapping brings the *PLAG1* gene under the transcriptional control of a more active promoter gene such as *COL3A1* and *CHCHD7* [9]. These genetic abnormalities in *PLAG1* help distinguish LPBs from other lipomatous neoplasms, although a subset of LPBs express polysomy of chromosome 8 or *HMGA2* gene rearrangements with or without *PLAG1* involvement. New fusion transcripts are being found associated with LPBs, including *MEG3* and *COL1A1* [10].

Here we review the current literature of LPBs and report on a three-month-old male who presented with a slowly enlarging, solitary, soft tissue tumor in the left posterolateral chest wall. The patient did not have any syndromic findings. The pregnancy was reported as uncomplicated, and the baby was full term at birth. There was no family history of genetic or congenital disorders. Ultrasound of the chest found a solitary 2.9 × 1.2 × 3 cm mass in the left posterolateral chest wall without color flow, touching adjacent ribs and pushing muscles anteriorly. Follow up magnetic resonance imaging (MRI) one month later demonstrated doubling in the size of the mass to 6.0 × 3.4 × 5.1 cm. The mass was surgically removed two months later. The specimen was submitted for pathology examination, and a portion was taken for cytogenetic analysis. The post-operative course was unremarkable with no further follow-up.

## Case presentation

### Pathology

The resected masses were all submitted fresh to Pathology and fresh unfixed tissue was taken for cytogenetic analysis before fixation of the tumor in 10% neutral buffered formalin. Sections were taken for routine processing. Immunohistochemistry was performed on formalin-fixed, paraffin-embedded tissues using standard techniques.

### Cytogenetics/cytogenomics

Chromosome analysis was performed as per standard protocol. Metaphase fluorescence in situ hybridization (FISH) was performed using TelVysion 8p Spectrum Green and TelVysion 8q Spectrum Orange (Abbott Molecular), and *MYC* break apart (BA) and CEP 8 probes (Abbott Molecular). In addition, interphase FISH was performed using a custom *PLAG1* BA probe combined with a CEP8, 3' *PLAG1* (centromeric-red), *5*' *PLAG1* (telomeric-green). A high-resolution array comparative genomic hybridization (aCGH) platform using Agilent’s 2 × 400 k CGH + SNP GenetiSure Cancer array (Agilent Technologies, Santa Clara, CA) was performed on DNA extracted from formalin-fixed paraffin embedded (FFPE) tissue as previously described (Zimran, E. et al. Haematologica, 2018). Copy number aberrations (CNAs) were filtered to exclude those < 100 kb, nested aberrations, Y chromosome calls in females, and reference DNA CNVs. Regions of CNLOH were called if they contained a minimum of 10 probes and were > 10 Mb in size.

## Results

**Fig. 1:**
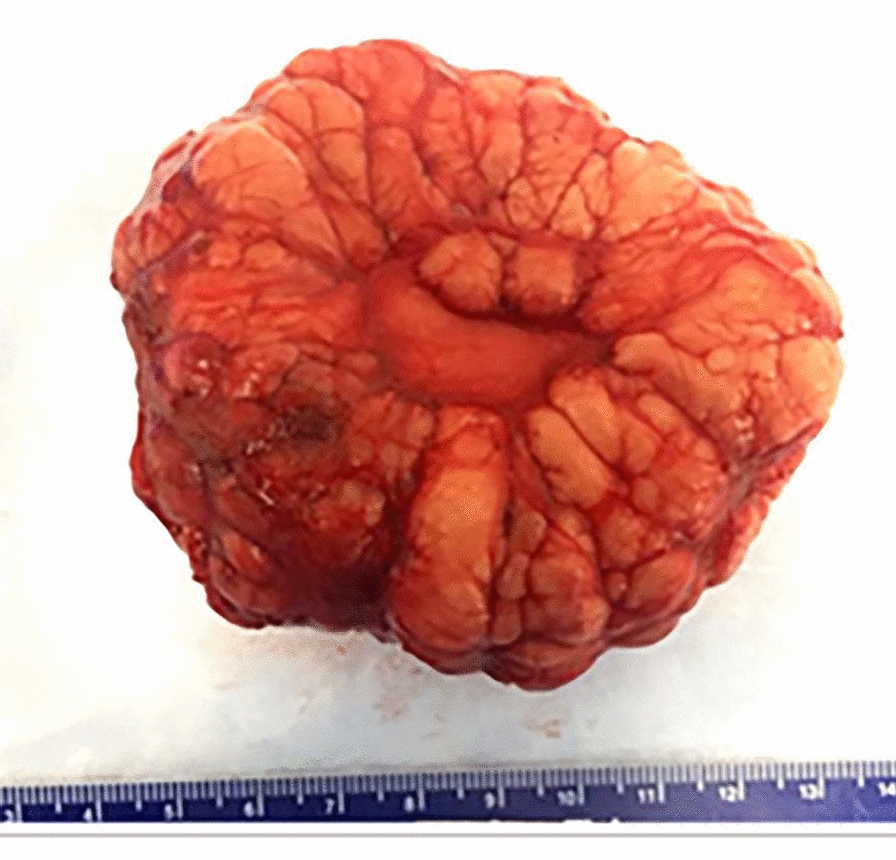
Gross picture of lobulated adipocytic tumor, later confirmed to be a lipoblastoma

**Fig. 2:**
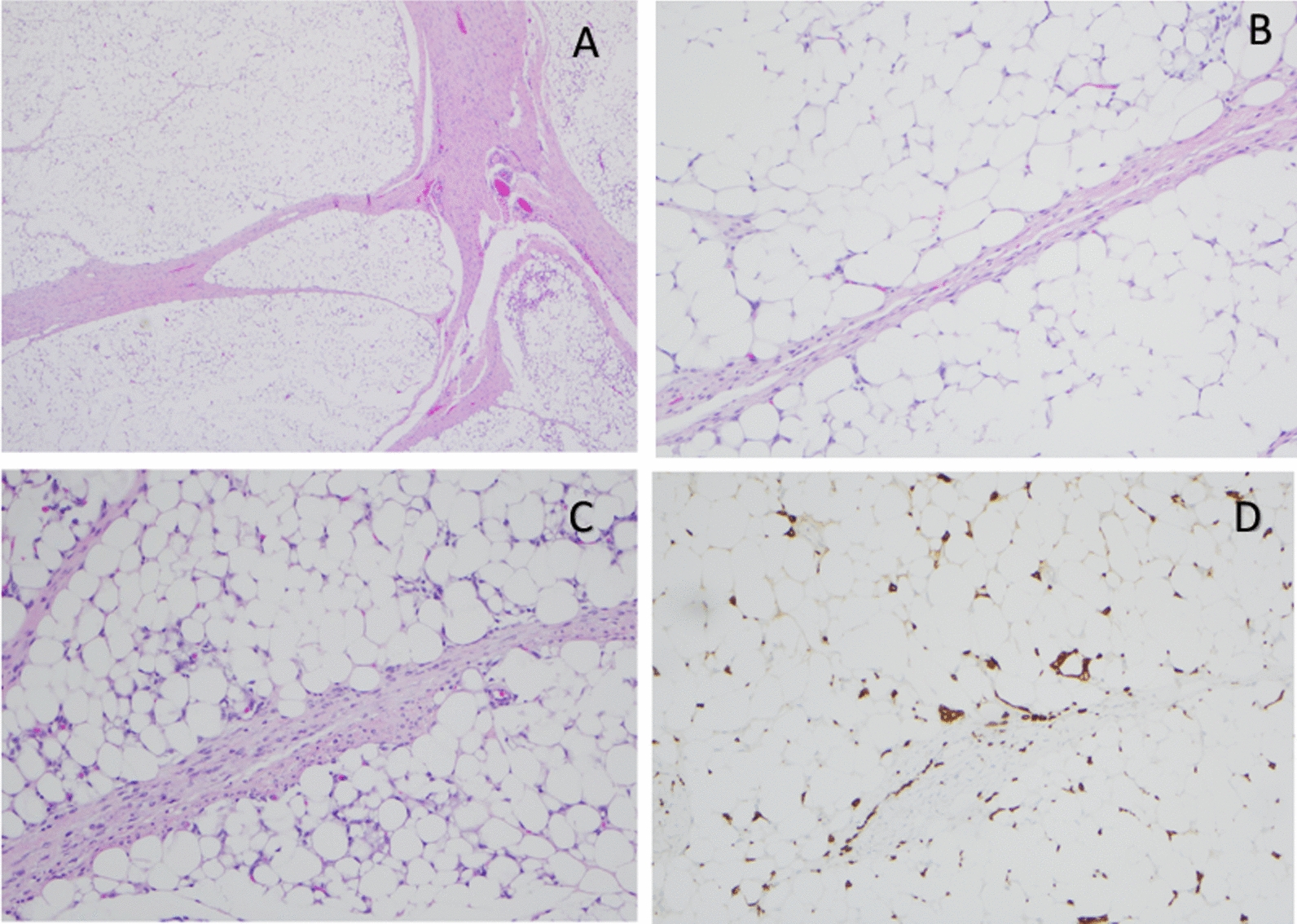
**A** H&E (20x): Lobular architecture with fibrous septa and lobules of mature adipose tissue. **B** H&E (20x): Fibrous septum and mature adipose tissue. No myxoid component and no spindle cell proliferation present. **C** H&E (20x): Fibrous septum and mature adipose tissue. Scattered macrophages present. **D** H&E (100x): CD163 immunoreactive macrophages present

The resected mass was a disc-shaped well circumscribed bosselated fatty mass with a delicate fibromembranous capsule (Fig. 1). Histology showed a prominent lobular pattern with mature adipose tissue, many intervening fibrous septa of variable thickness, and absence of any myxoid component (Fig. 2A, B). Foci of fat necrosis were present (Fig. 2C) and showed scattered CD163 immunoreactive macrophages (Fig. 2D).

**Fig. 3:**
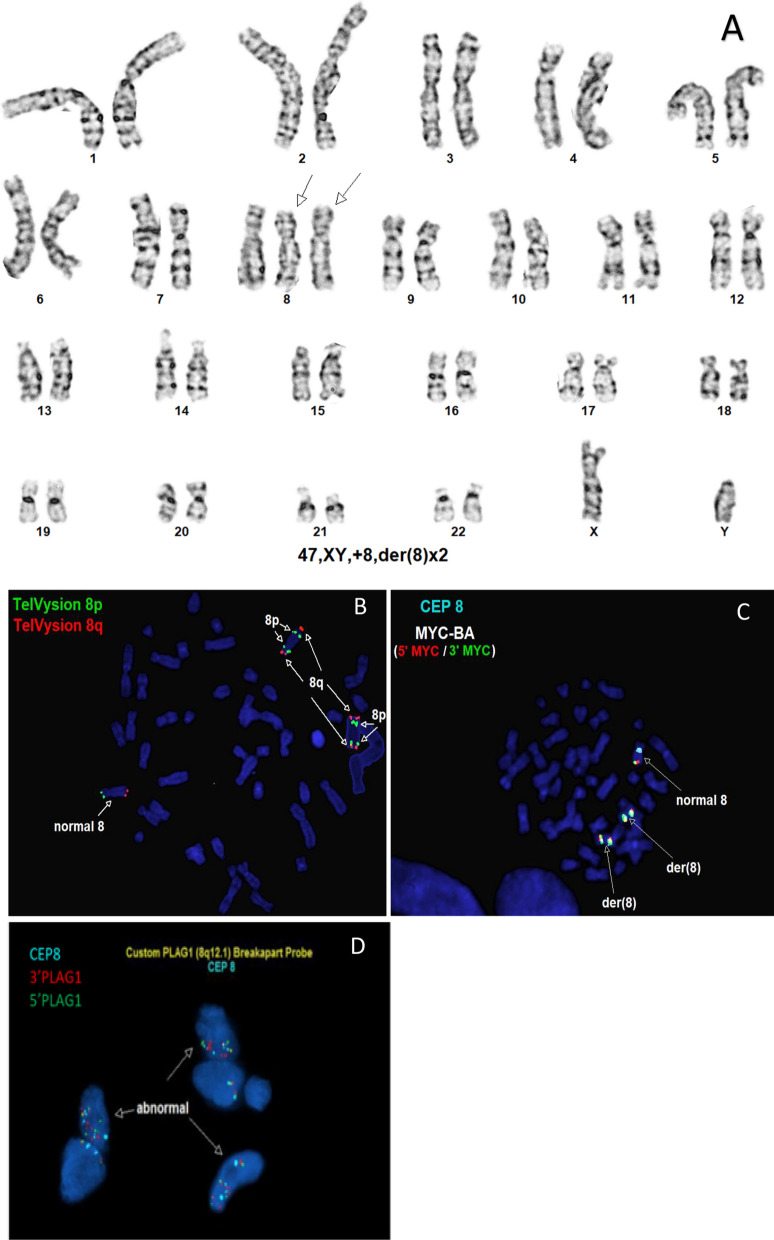

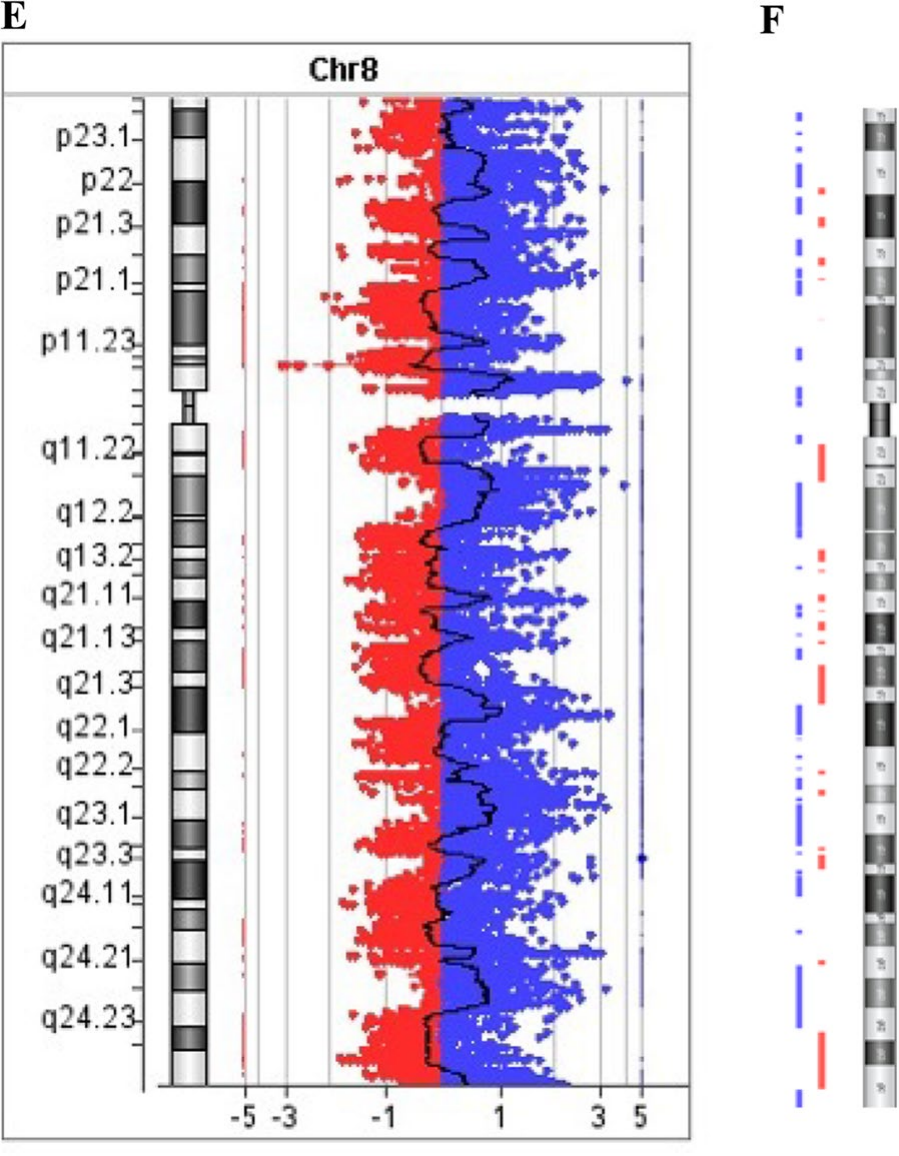
**A** Karyotype showing a 47,XY, + 8,der(8) × 2. **B** Metaphase FISH analysis with TelVysion 8p (green) and TelVysion 8q (orange) showing signals of 8p and 8q on the terminal ends of the short and the long arms on both abnormal der(8) chromosomes. **C** Metaphase FISH analysis with CEP8 (aqua) and *MYC* BA (green–red) probe showing all three signals present on both ends of the abnormal der(8) chromosomes. **D** FISH analysis on FFPE showing multiple copies of 3’ *PLAG1* (centromeric-red), 5’*PLAG1* (telomeric-green) and CEP8 (aqua). **E **Vertical CGH plot showing alternating 39 gains and 19 losses on chromosome 8. **F** Vertical plot of CGH with similar gains and losses. Blue denotes gain; Red denotes loss

Chromosome analysis revealed an abnormal karyotype with two abnormal derivative chromosomes 8 in addition to a normal copy of chromosome 8 described as 47,XY, + 8,der(8) × 2[10]/46,XY[10] (Fig. 3A). FISH analysis using chromosome 8p and 8q probes revealed abnormal hybridization patterns on both der[8] chromosomes with signals for 8p and 8q probes on both ends of each der(8) (Fig. 3B). FISH results using the *MYC* BA and CEP 8 probes confirmed the abnormal nature of the two der(8) chromosomes (Fig. 3C). FISH analysis on FFPE with a custom *PLAG1* BA probe and CEP8 showed multiple copies of each probe (Fig. 3D). Microarray analysis revealed 50 regions of alternating CNAs occurring on chromosome 8 (39 gains and 19 losses) (Fig. 3E, F). The average size of the CNAs was 1.9 Mb ranging from 17.8 kb to 9.2 Mb. Based on these combined findings, this tumor exhibits chromothripsis of chromosome 8 and is described as arr(8)cth. No other chromosomes were involved in the process of chromothripsis. This is the second known report of chromothripsis described in a benign LPB.

## Discussion and conclusions

LPBs were first described in 1926 as a tumor of immature adipose [11], but did not gain acceptance as a separate tumor entity until Vellioz et al. in 1958 described the neoplasm in relation to lipoblastomatosis.3 Microscopically, the excised tumor is composed of sheets of mature adipocytes arranged in a lobular architecture, intermixed with other adipocyte cells in varying stages of maturation. Literature review showed that LPBs are further subdivided into three histologic categories: a classic subtype composed primarily of embryonal white fat [12], a mature adipocytic subtype [12], and a myxoid subtype showing a plexiform vascular network with thin fibrous septa and pools of myxoid matrix [4, 5, 12].

Adipocytic tumors overall are exceptionally rare in children under the age of 10 years old. Over 80% of LPBs are diagnosed in children before three years of age with some present at birth and may show a male predilection [13–16]. The most affected regions are subcutaneous tissue of the extremities, but other locations include the head and neck [8], pelvis [17, 18], mediastinum [8, 19], mesentery [20], axilla [21], and abdomen [6, 19, 22]. A subset of patients diagnosed with LPB have developmental delays, seizures, familial lipoma syndromes, and congenital malformations such as cleft lip, cleft palate, cephalic malformations, seizures [1, 12, 23]. The prognosis after surgical removal is excellent, with no reported cases of malignant transformation. Follow-up is recommended for 5–10 years post-resection, as the recurrence rate for LPB is estimated to be 13–46% due to positive surgical margins, incomplete tumor resection, or lipoblastomatosis in the patient [5, 18].

The molecular hallmark of LPB is chromosomal alterations involving the *PLAG1* gene, although the exact pathogenesis remains unknown. [9] The current literature estimates that 60–70% of LPBs have a simple, aneuploid or hyperdiploid karyotype with a structural alteration in the 8q11–13 region, leading to the *PLAG1* rearrangement [1, 4, 5, 24, 25]. *PLAG1* encodes a zinc finger proto-oncogene with two putative nuclear localization signals. The majority of LPBs have identified *PLAG1* upregulation through promotor swapping with a more active promotor of the fusion genes, the most common being *HAS2* and *COL1A2* [13]. The number of possible fusion transcripts described in lipoblastomas have increased considerably over the past decade, as *SRSF3, HNRNPC, PCMTD1, YWHAZ, CTDSP2, PPP2R-2A, COL3A1, MEG3, RAD51B, BOC*, and two genes neighboring *PLAG1, RAB2A* and *CHCHD7*, have been identified as potential fusion partners [5, 8–10, 24, 24–29]. LPBs are immunohistochemically negative for *MDM2* and *CDK4* expression, as compared to well differentiated liposarcomas which are *MDM2* and *CDK4* positive with no associated clonal rearrangements of *PLAG1* [4].

Our patient showed chromosome 8 abnormalities with typical histologic findings. Some larger, multi-institutional studies have focused on the morphologic and immunophenotypic features of lipoblastomas [1, 30], while other series have looked at the molecular characteristics [9, 12]. The case presented in this report is unique in that microarray analysis clearly revealed evidence of chromothripsis. The overexpression of *PLAG1* suspected by FISH analysis was confirmed by the pronounced clustering of breakpoints and the oscillating copy-number profiles derived from the microarray supporting chromothripsis at 8q11-13 being responsible for the oncogenic process seen in this tumor.

Recently identified as an independent genetic phenomenon through genomic sequencing, chromothripsis results in massive chromosomal rearrangements and multiple downstream genomic aberrations [31–38]. While the underlying mechanisms resulting in chromothripsis are mostly unknown, chromothripsis has been associated with *TP53* mutations in subsets of medulloblastoma and acute myeloid leukemia [39–41]. The status of *TP53* was not tested for our patient and is unknown.

Although initial estimates of chromothripsis in tumors were low, there is growing evidence that it is associated with the genetic oncogenesis across a wider spectrum of cancers than initially believed, as more tumors are genetically sequenced [40, 42]. Chromothripsis has been detected in a diverse range of solid tumors, and hematological malignancies, resulting in aggressive tumor behavior and poor diagnostic outcomes [42–46]. Typically, *PLAG1* is activated in lipoblastoma and coincides with low-level amplification, (47) while in our case chromothripsis results in high level amplification in chromosome 8, while no other chromosomes were involved.

Chromothripsis has only once been previously reported in a lipoblastoma in the thigh of a 5-year-old male. Array CGH + SNP analysis of that LPB found multiple rearrangements localized on the long arm of chromosome 8 and pronounced clustering of breakpoints detected on chromosomal analysis [37]. Our patient and this previously reported case both show chromothripsis without *PLAG1* rearrangement, which suggests that chromothripsis is an alternative oncogenic mechanism in LPBs when no *PLAG1* fusions can be detected.

To the best of our knowledge this is only the second known report of chromothripsis described in a benign LPB and these findings broaden our understanding of the varied cytogenetic events in LPBs. Additional molecular studies of LPB are needed to understand the pathogenesis of this unusual tumor.

## Abbreviations

LPB: Lipoblastoma
FISH: Fluorescence in situ hybridization
*PLAG1*: Pleomorphic adenoma gene 1
BA: Break apart
aCGH: Array comparative genomic hybridization
CAN: Copy number aberration
